# Extended Venous Thromboembolism Prophylaxis After One-Anastomosis Gastric Bypass: A Three-Arm Randomized Trial of Enoxaparin Duration and Rivaroxaban

**DOI:** 10.1007/s11695-026-08882-0

**Published:** 2026-08-11

**Authors:** Mohammed Elshwadfy Nageeb, Nagy Salah Ismail, Abdelkarem Ahmed Abdelkarem Mohamed, Mohamed Yacoub, Khaled Mosleh Torfa, Mohamed Nasr Shazly

**Affiliations:** 1 General Surgery Department, Kasr Alainy Faculty of Medicine, Cairo University, Giza, Egypt; 2 Diagnostic and Interventional Radiology Department, , Kasr Alainy Faculty of Medicine, Cairo University, Giza, Egypt

**Keywords:** Bariatric surgery, Venous thromboembolism, Thromboprophylaxis, Low molecular weight heparin, Rivaroxaban, Direct oral anticoagulant, Extended prophylaxis

## Abstract

**Background:**

Venous thromboembolism (VTE) is a serious but preventable complication after bariatric surgery, most of them after hospital discharge. The optimal regimen and duration of post-discharge prophylaxis, particularly the role of direct oral anticoagulants, remain uncertain.

**Objectives:**

To estimate 30-day VTE and bleeding event rates in high-risk patients undergoing one-anastomosis gastric bypass who received 15-day enoxaparin, 30-day enoxaparin, or 30-day rivaroxaban prophylaxis, and to perform exploratory comparisons among the regimens.

**Methods:**

In this randomized, open-label, three-arm clinical trial, high-risk adults undergoing laparoscopic OAGB were randomized before discharge (1:1:1) to enoxaparin 40 mg subcutaneously twice daily for 15 days, enoxaparin 40 mg twice daily for 30 days, or rivaroxaban 10 mg orally once daily for 30 days, after standardized in-hospital enoxaparin and early ambulation. Participants underwent clinical assessment and duplex ultrasonography of the lower-limb and porto-mesenteric veins on postoperative days 15 and 30. The primary outcome was objectively confirmed VTE within 30 days. Bleeding was classified as International Society on Thrombosis and Haemostasis (ISTH) major bleeding or clinically relevant non-major bleeding (CRNMB). Because the expected event rate was low and no non-inferiority or equivalence margin was prespecified, comparisons were interpreted as exploratory.

**Results:**

A total of 288 patients were randomized to 15-day enoxaparin (*n* = 97), 30-day enoxaparin (*n* = 97), or 30-day rivaroxaban (*n* = 94). One symptomatic lower-limb deep vein thrombosis occurred in the 15-day enoxaparin group (1.0%; 95% CI, 0.03%–5.6%); no VTE events occurred in the 30-day enoxaparin group (0%; 95% CI, 0%–3.7%) or the rivaroxaban group (0%; 95% CI, 0%–3.8%). Total bleeding occurred in 4/97 patients (4.1%) in each enoxaparin group and 8/94 patients (8.5%) in the rivaroxaban group. The absolute difference in total bleeding between rivaroxaban and 30-day enoxaparin was 4.4% points (95% CI, − 2.9 to 12.2), indicating substantial imprecision. No porto-mesenteric venous thrombosis was detected.

**Conclusion:**

Only one VTE event occurred, precluding reliable conclusions regarding comparative efficacy or prophylaxis duration. Bleeding estimates were also imprecise and do not establish comparative safety or equivalence between rivaroxaban and enoxaparin. The trial adds descriptive event-rate data from a standardized OAGB pathway, but larger multicenter studies with prespecified comparative hypotheses and assessment of adherence, oral tolerance, and drug exposure are required.

The study was approved by the Research Ethics Committee and registered at ClinicalTrials.gov.

## Key Points

Prophylaxis for VTE after bariatric surgery lacks standardized regimens.

High-risk gastric bypass patients received extended thromboprophylaxis.

Modern OAGB pathways were associated with a very low observed 30-day VTE rate.

Only one VTE event occurred among 288 participants, preventing reliable comparative efficacy conclusions.

Bleeding estimates were imprecise and did not establish similar safety between rivaroxaban and enoxaparin.

The study did not determine whether 15 days is sufficient or whether rivaroxaban is equivalent or non-inferior to LMWH.

## Introduction

Venous thromboembolism (VTE) is a notable and preventable complication following metabolic–bariatric surgery, with a 30-day postoperative incidence reported between 0.2% and 1.3% [1–3]. Events such as pulmonary embolism carry a significant risk of rapid mortality. Although the overall incidence of VTE in this patient population is relatively low, complications like deep vein thrombosis and portal-mesenteric venous thrombosis (PMVT) can result in substantial morbidity and often require extended anticoagulation therapy. People with obesity are predisposed to a prothrombotic state, influenced by factors such as obesity-related hypercoagulability, venous stasis, chronic inflammation, and comorbid cardiometabolic conditions [4–6]. These risks can be compounded by the perioperative surgical stress response [7, 8], necessitating vigilant assessment and prevention strategies to mitigate VTE occurrence in this cohort.

Notably, many VTE incidents post-bariatric surgery occur after patients are discharged from the hospital, reflecting a critical aspect of perioperative risk management [3]. Although modern minimally invasive surgical techniques and optimized perioperative pathways have reduced hospitalization times, the biological predisposition for thrombosis persists for several weeks postoperatively. Factors such as temporary reduced mobility, dehydration, inadequate oral intake, and sustained postoperative inflammatory responses may prolong this risk, particularly into the third and fourth weeks following surgery [8, 9]. This temporal alignment underscores the rationale for considering extended pharmacologic thromboprophylaxis in select high-risk individuals.

Low molecular weight heparin (LMWH) has historically been the cornerstone of thromboprophylactic strategies in bariatric surgery, often extended beyond inpatient care [10]. Pharmacologically, LMWH potentiates antithrombin activity, leading to predominant inhibition of factor Xa and, to a lesser extent, thrombin (factor IIa), thereby suppressing clot propagation in a predictable, weight-adjustable manner [11, 12]. Nonetheless, the ideal duration for outpatient LMWH remains indeterminate, as prolonged injection protocols can undermine adherence [13], elevate costs, and induce localized adverse reactions. Direct oral anticoagulants (DOACs) present an alternative with fixed dosing regimens that may enhance patient acceptability [14]; agents such as rivaroxaban act through direct, selective inhibition of factor Xa, blocking conversion of prothrombin to thrombin without reliance on antithrombin [15]. However, concerns linger regarding the altered pharmacokinetics and absorption of orally administered DOACs following bariatric interventions and the limited availability of robust comparative clinical outcome data in this population [16–18].

Following gastric bypass procedures, DOAC exposure may be affected by altered gastric pH, reduced absorptive surface, and bypass of proximal small-bowel segments, creating theoretical concerns for under-absorption—particularly for agents with predominant upper gastrointestinal uptake. Accordingly, early clinical guidance and subsequent evidence syntheses advised caution with DOAC use after bariatric surgery and favored parenteral anticoagulation or warfarin when reliable long-term oral anticoagulation is required [17, 19]. However, emerging pharmacokinetic data and clinical outcome studies have been more reassuring, and a randomized trial of rivaroxaban thromboprophylaxis in post-bariatric surgery patients reported low VTE rates with acceptable safety using fixed-dose regimens [19, 20]. Despite these signals, procedure-specific data for bypass operations remain limited, and careful patient selection remains essential [21].

In terms of treatment duration, the debate persists. A two-week regimen may offer simplicity and cost-effectiveness, yet it risks overlooking the delayed post-discharge VTE window. Conversely, a four-week course may align more closely with the sustained postoperative biological risk profile but could increase cumulative bleeding exposure. Hence, evaluating the safety of oral prophylaxis as a replacement for extended LMWH and establishing the adequacy of shorter LMWH courses remains a pressing clinical question.

Accordingly, this randomized three-arm trial enrolled high-risk patients undergoing OAGB and evaluated three post-discharge prophylaxis strategies: enoxaparin for 15 days, enoxaparin for 30 days, and rivaroxaban for 30 days. The primary objective was to estimate the incidence of objectively confirmed postoperative VTE within 30 days in each group. Secondary objectives were to estimate ISTH major bleeding, CRNMB, and allergic reactions. Because no non-inferiority or equivalence margin was prespecified, between-group comparisons were considered exploratory.

## Methods

### Study Design and Setting

This prospective randomized clinical trial evaluated extended venous thromboembolism prophylaxis after laparoscopic one-anastomosis gastric bypass (OAGB). The study was carried out in the Department of General Surgery, University Hospitals, where patients undergoing One Anastomosis Gastric Bypasswere recruited and followed.

## Participants and Eligibility Criteria

Patients undergoing laparoscopic one anastomosis gastric bypasswere eligible if they were considered at high risk for postoperative VTE. High-risk status was defined as a calculated postoperative VTE score > 0.4% according to the Cleveland Clinic Risk Calculator (Aminian model) [22] and/or the presence of recognized clinical risk factors, including a previous VTE event, immobility, hypercoagulable conditions, venous stasis disease, post-menopausal hormonal therapy, or oral contraceptive use. Patients were excluded if they were pregnant or lactating, had active substance or alcohol abuse, active gastric ulcer disease, psychological instability that could impair adherence, or proven preoperative VTE.

## Baseline Assessment

For all enrolled patients, demographic and clinical variables were documented, including age, sex, body mass index, associated comorbidities, and any intraoperative or postoperative complications, with particular attention to thromboembolic and bleeding events. To exclude preexisting thrombosis, all participants underwent bilateral lower-limb venous duplex ultrasound and porto-mesenteric (mesenteric/portal) duplex ultrasound within one week prior to surgery.

## Perioperative Thromboprophylaxis

All patients received standardized perioperative mechanical and pharmacologic prophylaxis with enoxaparin 40 mg subcutaneously, administered 12 h before surgery andrepeated 6 h postoperatively then every 12 h until the hospital discharge.

## Randomization and Postoperative Prophylaxis Regimens

Before hospital discharge, eligible patients were randomized in a 1:1:1 ratio using a computer-generated allocation sequence prepared by an independent team member not involved in recruitment or outcome assessment. Allocation concealment was ensured using sequentially numbered opaque sealed envelopes (or an equivalent concealment method), opened only after eligibility confirmation. Patients were assigned to one of three extended prophylaxis groups: (1) enoxaparin 40 mg subcutaneously twice daily for 30 days, (2) rivaroxaban 10 mg orally once daily for 30 days, or (3) enoxaparin 40 mg subcutaneously twice daily for 15 days.

### Follow-up and Surveillance

Patients were followed clinically for signs or symptoms of VTE for three months. Duplex ultrasound surveillance of the lower-limb venous system and porto-mesenteric venous circulation was performed postoperatively to detect VTE, with a mandatory assessment at 15 and30 days after surgery. Any clinically suspected thromboembolic event before this time point prompted immediate evaluation according to standard unit practice.

## Outcomes

The primary outcome was the number of postoperative VTE events within 30 days, including lower-limb VTE and porto-mesenteric venous thrombosis, in each treatment group. Secondary outcomes were the number of bleeding events and allergic reactions attributable to the prophylactic regimen.Participants were followed clinically for thrombotic and bleeding symptoms for 30 days. Scheduled lower-limb and porto-mesenteric duplex ultrasonography was performed on postoperative days 15 and 30. Suspected events prompted immediate investigation. The study did not formally measure outpatient adherence using pill counts, injection diaries, electronic monitoring, or pharmacy refill data. It did not systematically record missed or vomited doses, nausea, vomiting, poor oral intake, or dehydration in relation to medication administration, and rivaroxaban or anti-factor Xa levels were not measured.

## Sample Size Calculation

Because this protocol deliberately targeted high-risk patients rather than an unselected bariatric population, sample-size planning considered contemporary VTE incidence estimates and risk-enriched strata, in which postoperative VTE risk may be substantially higher than average (up to 3%) and has been reported to approach the upper single-digit per-cent range in some series [1–3], and is explicitly quantified using the Aminian post-discharge risk tool [22]. Sample size was determined using P.S. Power 3.1.6 based on a reference postoperative thrombosis probability of 0.007. With a two-sided α of 0.05 and 80% power, the required sample size was 93 patients per group.

### Statistical Analysis

Statistical analysis was performed using IBM SPSS Statistics for Windows, Version 25.0 (IBM Corp., Armonk, NY). Continuous variables were assessed for normality using the Shapiro–Wilk test and presented as mean ± standard deviation (SD) if normally distributed or as median (interquartile range, IQR) if non-normally distributed, while categorical variables were expressed as frequency and percentage. Comparisons among the three study groups (LMWH 2 weeks, LMWH 4 weeks, and DOAC 4 weeks) were conducted using one-way analysis of variance (ANOVA) for normally distributed continuous variables, followed by Bonferroni post-hoc test for pairwise differences, and the Kruskal–Wallis test for non-normally distributed variables. Categorical variables were compared using the Chi-square (χ²) test. All statistical tests were two-tailed, and a p-value < 0.05 was considered statistically significant.

## Results

### Participant Flow and Allocation

**Fig. 1:**
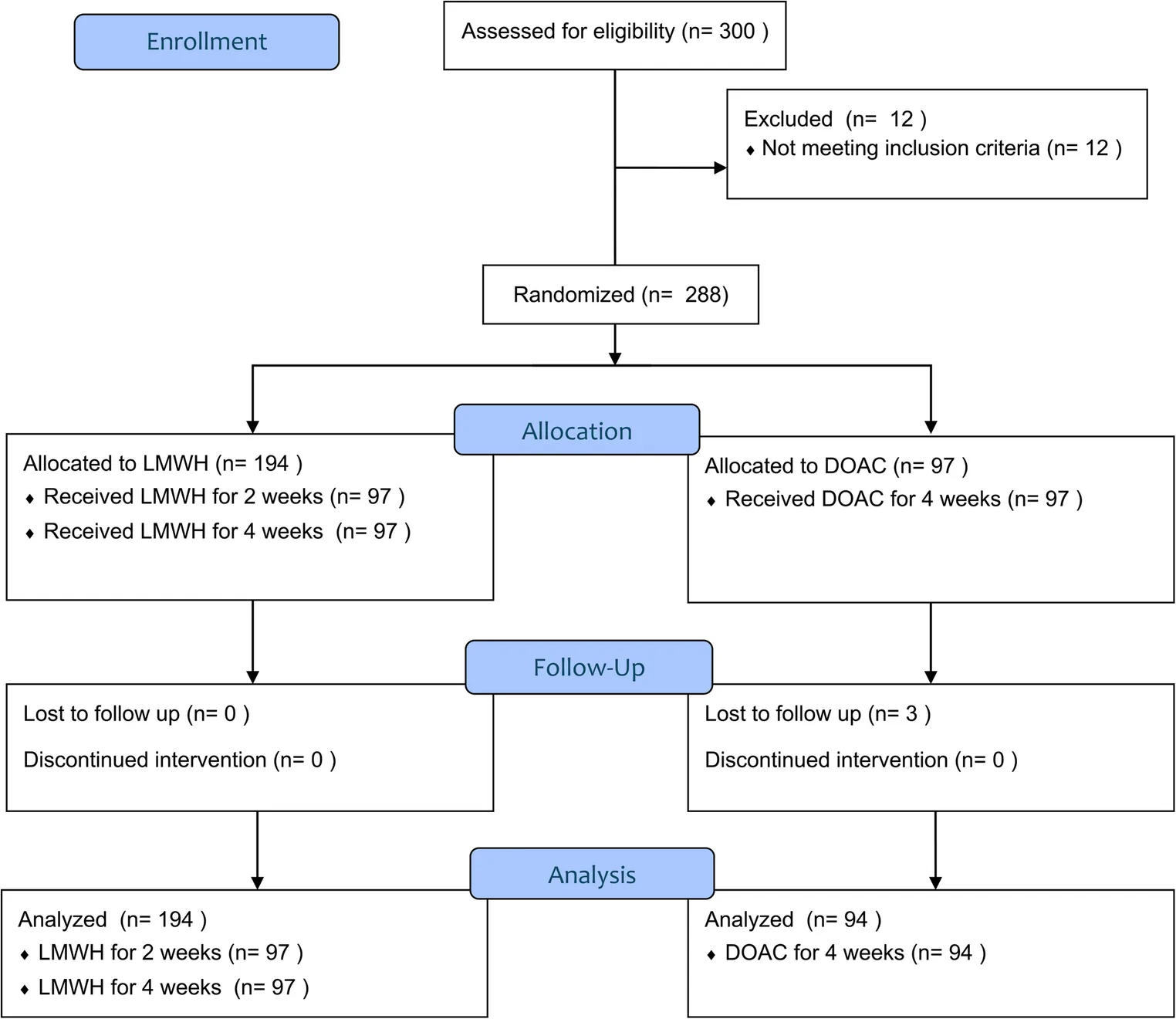
CONSORT flow diagram of enrollment, randomization, allocation, follow-up, and analysis

A total of 288 eligible patients undergoing laparoscopic one anastomosis gastric bypasswere randomized (1:1:1) to LMWH for 2 weeks (*n* = 97), LMWH for 4 weeks (*n* = 97), or direct oral anticoagulant (DOAC), rivaroxaban, for 4 weeks (*n* = 94). All randomized participants received the allocated intervention and were included in the primary analysis (intention-to-treat), with clinical and duplex ultrasound follow-up completed through postoperative day 30 (Fig. 1).

### Baseline Characteristics

A total of 288 patients were included in the study, distributed equally among the three treatment groups: LMWH for 2 weeks (*n* = 97), LMWH for 4 weeks (*n* = 97), and DOAC (rivaroxaban) for 4 weeks (*n* = 94).

Baseline demographic characteristics and comorbidities are shown in Table 1. The three randomized groups had broadly similar distributions of age, sex, body mass index, smoking status, and recorded comorbidities. The baseline P values are presented descriptively and were not used to establish the success of randomization or to infer treatment effects.

**Table 1:** Baseline demographic and preoperative comorbidities

	LMWH 2 weeks	LMWH 4 weeks	DOAC 4 weeks	Test statistic	*P*-value
	(*n* = 97)	(*n* = 97)	(*n* = 94)		
Age, year ^a^	36 (28–41)	35 (28–41)	37 (28–42.25)	K = 1.351	0.509
Sex Female Male	77 (79.4) 20 (20.6)	78 (80.4) 19 (19.6)	81 (86.2) 13 (13.8)	𝒳^2^ = 1.719	0.423
Weight ^b^	131.71 ± 16.11	131.18 ± 16.59	126.86 ± 19.9	F = 2.170	0.116
BMI ^b^	49.36 ± 5.57	49.22 ± 5.72	48.17 ± 7.37	F = 1.021	0.362
Smoking status	6 (6.2)	6 (6.2)	6 (6.4)	𝒳^2^ = 0.004	0.998
Hypertension	17 (17.5)	15 (15.5)	18 (19.1)	𝒳^2^ = 0.455	0.797
Diabetes Mellitus	10 (10.3)	13 (13.4)	11 (11.7)	𝒳^2^ = 0.447	0.800
OSAS	7 (7.2)	6 (6.2)	5 (5.3)	𝒳^2^ = 0.294	0.863
Ischemic heart disease	2 (2.1)	2 (2.1)	3 (3.2)	𝒳^2^ = 0.341	0.843
Dyslipidemia	6 (6.2)	6 (6.2)	6 (6.4)	𝒳^2^ = 0.004	0.998
Immobility	3 (3.1)	3 (3.1)	1 (1.1)	𝒳^2^ = 1.099	0.577
Asthmatic Previous DVT(all post CS)	3 (3.1) 1 (1)	4 (4.1) 2 (2.1)	1 (1.1) 2 (2.1)	𝒳^2^ = 1.709 𝒳^2^ = 0.428	0.426 0.808
Previous Surgeries					
Cesarean section Ureteric lithotripthy Lab Appendectomy Open Appendectomy Tonsillectomy Open Cholecystectomy Lap Cholecystectomy Hernioplasty Lap. ovarian cystectomy	34 (35.1) 2 (2.1) 3 (3.1) 2 (2.1) 2 (2.1) 2 (2.1) 1 (1) 2 (2.1) 0 (0)	33 (34) 2 (2.1) 4 (4.1) 2 (2.1) 2 (2.1) 2 (2.1) 2 (2.1) 2 (2.1) 0 (0)	30 (31.9) 0 (0) 1 (1.1) 5 (5.3) 6 (6.4) 3 (3.2) 0 (0) 2 (2.1) 2 (2.1)	𝒳^2^ = 0.218 𝒳^2^ = 1.965 𝒳^2^ = 1.709 𝒳^2^ = 2.219 𝒳^2^ = 3.528 𝒳^2^ = 0.341 𝒳^2^ = 1.969 𝒳^2^ = 0.001 𝒳^2^ = 4.157	0.897 0.374 0.426 0.330 0.171 0.843 0.374 0.999 0.125

CS, cesarean section

All values presented in Number (percentage) unless indicated otherwise

^a^ median (Q1-Q3)

^b^mean± SD.

### Preoperative and Intraoperative Variables

Preoperative duplex ultrasonography confirmed that all participants were free of venous thrombosis before surgery. Other preoperative and intraoperative variables, including operative time, are reported in Table 2. These variables preceded post-discharge treatment allocation and are presented for description of the randomized groups.

**Table 2:** Preoperative and intraoperative variables

	LMWH 2 weeks	LMWH 4 weeks	DOAC 4 weeks	Test statistic	*P*-value
	(*n* = 97)	(*n* = 97)	(*n* = 94)		
Preoperative duplex Free	97 (100)	97 (100)	94 (100)	-	-
Operative time, hours ^a^	2 (1.5–2.5)	2 (1.5–2.25)	2 (1.5–2.5)	K = 0.180	0.914

All values presented in Number (percentage)

^a^ median (Q1-Q3)

### Postoperative Findings

No lower-limb or porto-mesenteric venous thrombosis was detected at the scheduled day-15 duplex assessment. One patient in the 15-day LMWH group developed clinically suspected lower-limb DVT on postoperative day 22, which was confirmed by duplex ultrasonography; no DVT was recorded in the other groups. Major bleeding was recorded in 1 patient in each LMWH group and 2 patients in the rivaroxaban group. Events recorded as minor bleeding occurred in 3, 3, and 6 patients, respectively, and total recorded bleeding occurred in 4, 4, and 8 patients, respectively. The corresponding P values were not statistically significant, but the event counts were too small for non-significance to establish equivalence or comparable safety. Other postoperative complications are reported in Table 3.

**Table 3:** Postoperative outcomes

	LMWH 2 weeks	LMWH 4 weeks	DOAC 4 weeks	Test statistic	*P*-value
	(*n* = 97)	(*n* = 97)	(*n* = 94)		
Hospital stay, days ^a^	2(2–2)	2 (2–2)	2(2–2)	-	-
Postoperative venous duplex day 15	0 (0)	0 (0)	0 (0)	-	-
Postoperative venous duplex day 15	1 (1)	0 (0)	0 (0)	𝒳^2^ = 1.976	0.372
Major bleeding ^ISTH criteria^	1 (1)	1 (1)	2 (2.1)	𝒳^2^ = 0.556	0.757
Minor bleeding ^ISTH criteria^	3 (3.1)	3 (3.1)	6 (6.4)	𝒳^2^ = 1.717	0.424
Total Bleeding	4 (4.1)	4 (4.1)	8 (8.5)	𝒳^2^ = 2.322	0.313
Early post operative complications					
Early post operative complications Pneumonia Port site infection Fever due to atelectasis Pleural effusion PVT Superficial thrombophlebitis	0 (0) 2 (2.1) 4 (4.1) 0 (0) 1 (1) 0 (0)	1 (1) 2 (2.1) 3 (3.1) 0 (0) 0 (0) 0 (0)	3 (3.2) 2 (2.1) 2 (2.1) 2 (2.1) 0 (0) 1 (1.1)	𝒳^2^ = 3.687 𝒳^2^ = 0.001 𝒳^2^ = 0.629 𝒳^2^ = 4.157 𝒳^2^ = 1.976 𝒳^2^ = 2.071	0.158 0.999 0.730 0.125 0.372 0.355

ISTH, the International Society on Thrombosis and Haemostasis

All values presented in Number (percentage) unless indicated otherwise

^a^ median (Q1-Q3)

## Discussion

### Principal Findings

This randomized three-arm trial observed one confirmed lower-limb DVT among 288 high-risk patients undergoing OAGB. The event occurred in the 15-day LMWH group after prophylaxis had ended, while no VTE event was recorded in the two 30-day groups. These observations are clinically reassuring, but one event is insufficient to determine whether prophylaxis duration or drug choice affected efficacy.

The low number of events may be more informative about the contemporary perioperative pathway than about differences between anticoagulant regimens. All patients underwent minimally invasive surgery, received standardized inpatient prophylaxis, were encouraged to mobilize early, and were followed closely. When the baseline event rate is this low, a study of this size cannot reliably distinguish modest but clinically important differences. Consequently, absence of statistical significance must not be interpreted as equivalence, non-inferiority, or proof of comparable safety.

### Comparison with Previous Literature

Population-based studies and large registries have reported low absolute VTE rates after bariatric surgery, with many events occurring after discharge [1–3]. The current study also observed a low event rate despite enrolling patients considered at increased risk. This finding is consistent with effective modern perioperative care, but it simultaneously limits the ability to draw comparative conclusions about the three prophylaxis strategies.

The single DVT occurred on postoperative day 22 after completion of the 15-day LMWH course. Although the timing is clinically noteworthy, it cannot demonstrate that a 15-day course is inadequate or that 30-day prophylaxis is superior. The event could reflect residual postoperative risk, but it could also represent chance. Therefore, the study does not determine the optimal duration of prophylaxis.

The rivaroxaban arm also requires cautious interpretation. Patients may experience nausea, vomiting, limited oral intake, or dehydration during the early postoperative period. These factors may affect whether an oral dose is taken and retained, while altered anatomy after OAGB raises additional uncertainty regarding absorption. The study did not formally assess adherence, missed or vomited doses, medication tolerance, or drug levels. Therefore, the absence of recorded VTE in the rivaroxaban group cannot confirm adequate exposure in every participant or establish equivalence with LMWH.

Existing pharmacokinetic and clinical studies provide some supportive evidence for rivaroxaban after bariatric surgery [17, 19, 20, 23, 24]. Nevertheless, differences in procedure type, timing, dose, and outcome frequency limit direct extrapolation. The present study contributes OAGB-specific clinical observations but should not be interpreted as proving that rivaroxaban is as effective as LMWH.

### Clinical Implications

The practical advantages and disadvantages of each route should also be considered. An oral regimen avoids prolonged injections, but its effectiveness depends on adherence, oral tolerance, and absorption. Injectable LMWH provides greater certainty that a successfully administered dose entered the body, although injections may cause discomfort and may be difficult for some patients to continue. Because adherence, tolerance, and patient-reported treatment burden were not measured, the study cannot determine which route was more acceptable or reliably delivered.

Bleeding outcomes were also too sparse for definitive safety conclusions. Total recorded bleeding occurred in 8 patients receiving rivaroxaban compared with 4 patients in each LMWH group. Although the between-group comparison was not statistically significant, this does not exclude a clinically important difference, including the possibility of more bleeding with rivaroxaban. Moreover, the study recorded bleeding as major or minor according to its original clinical categories; events were not formally adjudicated using the ISTH major bleeding and clinically relevant non-major bleeding framework. This limits comparison with other anticoagulation studies.

The numerical bleeding rates also do not capture all practical aspects of postoperative bleeding management. Interruption, reversal, reoperation, transfusion, and the ability to confirm the timing of the last effective dose may differ between LMWH and a direct oral anticoagulant. These management outcomes were not examined and should be considered when interpreting clinical feasibility.

Cost and access may further influence regimen selection. Drug price, insurance coverage, availability, the feasibility of self-injection, caregiver assistance, and access to emergency assessment or reversal strategies vary across healthcare systems. Because the study did not include a formal cost or access analysis, it cannot identify the most affordable or practical regimen.

### Strengths and Limitations

The randomized design, standardized perioperative pathway, focus on patients considered at increased VTE risk, and scheduled duplex surveillance are strengths. These features support a careful description of the observed events within this OAGB population.

The most important limitation is the very small number of efficacy and safety events. Only one VTE occurred, and the bleeding counts were also limited. The study therefore lacks sufficient information to establish comparative efficacy, comparative safety, equivalence, non-inferiority, or the optimal prophylaxis duration. The original sample-size calculation is retained as prespecified, but the much lower observed event frequency resulted in markedly limited comparative power.

The single-center and OAGB-only design also restricts generalisability. These findings may not apply to sleeve gastrectomy, Roux-en-Y gastric bypass, SADI-S, or biliopancreatic diversion with duodenal switch, because anatomy, drug absorption, postoperative oral intake, and recovery patterns differ. They may also not apply to older or more medically complex populations or to centers using different perioperative pathways.

The study did not formally measure adherence to oral or injectable prophylaxis, nausea or vomiting around medication administration, poor oral intake, dehydration, or anticoagulant drug levels. Accordingly, actual outpatient exposure could not be confirmed. Cost, access, patient preference, and the practical consequences of managing bleeding were also not evaluated.

The study used the original clinical categories of major and minor bleeding rather than formal ISTH adjudication. These categories should therefore not be assumed to correspond directly to ISTH major bleeding and clinically relevant non-major bleeding. The open-label design may also have influenced reporting of subjective events, while the low number of outcomes limits any assessment of such bias.

### Future Directions

Future studies will require substantially larger multicenter samples or registry-based randomized designs to distinguish clinically meaningful differences when VTE is uncommon. Comparative hypotheses and clinically acceptable margins should be prespecified when superiority, non-inferiority, or equivalence is intended.

Future work should also measure adherence, missed or vomited doses, postoperative oral tolerance, dehydration, patient preference, treatment burden, cost, and access. Standardized bleeding definitions and detailed reporting of management consequences would improve comparison across studies.

Until such evidence is available, the current results should be viewed as descriptive and hypothesis generating. They do not identify a preferred drug or duration, and prophylaxis decisions should continue to reflect individual thrombotic and bleeding risks, procedure type, patient circumstances, and local practice.

## Conclusion

In this randomized trial of high-risk patients undergoing laparoscopic OAGB, only one 30-day VTE event and a small number of bleeding events were recorded. These findings describe outcomes within a standardized contemporary pathway but do not establish comparative efficacy or safety, equivalence between rivaroxaban and LMWH, or adequacy of 15 versus 30 days of prophylaxis. Larger multicenter studies with more outcome events and assessment of adherence, oral tolerance, bleeding management, cost, and access are required.

## Data Availability

The datasets generated and analyzed during the current study are available from the corresponding author upon request.

## Abbreviations

ANOVA: analysis of variance
BMI: body mass index
DOAC: direct oral anticoagulant
DVT: deep vein thrombosis
IQR: interquartile range
LMWH: low molecular weight heparin
PMVT: porto-mesenteric venous thrombosis
SD: standard deviation
SPSS: Statistical Package for the Social Sciences
VTE: venous thromboembolism
